# Outlier Detection Transilience-Probabilistic Model for Wind Tunnels Based on Sensor Data

**DOI:** 10.3390/s21072532

**Published:** 2021-04-04

**Authors:** Encarna Quesada, Juan J. Cuadrado-Gallego, Miguel Ángel Patricio, Luis Usero

**Affiliations:** 1 Zitrón, S.A., 33211 Gijón, Spain; equesada@zitron.com; 2 Department of Computer Science, Universidad de Alcalá, Alcalá de Henares, 28805 Madrid, Spain; luis.usero@uah.es; 3 Department of Computer Science and Software Engineering, Concordia University, Montreal, QC H3G 1M8, Canada; 4 Department of Computer Science and Artificial Intelligence, Universidad Carlos III de Madrid, Colmenarejo, 28270 Madrid, Spain; mpatrici@inf.uc3m.es

**Keywords:** anomaly detection, ventilation systems, wind tunnels

## Abstract

Anomaly Detection research is focused on the development and application of methods that allow for the identification of data that are different enough—compared with the rest of the data set that is being analyzed—and considered anomalies (or, as they are more commonly called, outliers). These values mainly originate from two sources: they may be errors introduced during the collection or handling of the data, or they can be correct, but very different from the rest of the values. It is essential to correctly identify each type as, in the first case, they must be removed from the data set but, in the second case, they must be carefully analyzed and taken into account. The correct selection and use of the model to be applied to a specific problem is fundamental for the success of the anomaly detection study and, in many cases, the use of only one model cannot provide sufficient results, which can be only reached by using a mixture model resulting from the integration of existing and/or ad hoc-developed models. This is the kind of model that is developed and applied to solve the problem presented in this paper. This study deals with the definition and application of an anomaly detection model that combines statistical models and a new method defined by the authors, the Local Transilience Outlier Identification Method, in order to improve the identification of outliers in the sensor-obtained values of variables that affect the operations of wind tunnels. The correct detection of outliers for the variables involved in wind tunnel operations is very important for the industrial ventilation systems industry, especially for vertical wind tunnels, which are used as training facilities for indoor skydiving, as the incorrect performance of such devices may put human lives at risk. In consequence, the use of the presented model for outlier detection may have a high impact in this industrial sector. In this research work, a proof-of-concept is carried out using data from a real installation, in order to test the proposed anomaly analysis method and its application to control the correct performance of wind tunnels.

## 1. Introduction

The Ventilation Systems industry is focused on providing solutions for diverse sectors, such as mining, civil works, marine, and wind tunnel infrastructures, among others. For some of these sectors, ventilation is responsible for ensuring the safety of users and workers by guaranteeing oxygen levels and reducing the presence of dangerous gases or smoke. Therefore, the goal in these industries is to provide ventilation systems that are capable of meeting the demands of air flow, guaranteeing air quality, and achieving efficiency in the absence of system failures.

In the case of wind tunnel infrastructure—the object of study of this work—the fans that are part of the system are designed with different purposes, providing a concentrated stream of air in either a vertical or horizontal flow, in order to perform different tests on aircrafts [1], rocket models [2], and cars; to measure the aerodynamic of objects and systems [3]; to study fluid dynamics; and for the research and development of machines or buildings for which the flux of air around them is a critical matter [4]. Wind tunnels are also used as training facilities. Some types of wind tunnels, such as vertical wind tunnels for indoor skydiving applications, have the purpose of simulating air-flow characteristics similar to those a skydiver experiences in free-fall. Indoor skydiving systems were first introduced by the US military in the 1960s for training purposes. Today, indoor skydiving facilities are used to support military parachute training, to train skydivers, and also for the leisure industry [5].

In terms of the performance of the system in these types of facilities, the main goals of wind tunnels are the safety of the users and workers, energy efficiency, and the reduction of shutdowns due to system failure. The prevention of shutdowns of the system has become another important challenge to face, not just for these kind of facilities but, in general, for the Industry 4.0 paradigm, in which unplanned downtimes can lead to catastrophic consequences in terms of economic costs.

These systems require mechanisms to detect errors during functioning and to predict how much time is left before a system failure can occur. For this, it is necessary to continuously monitor and observe the operation of a system, thus detecting variations and deviations from the normal performance of the machines [6]. Therefore, current and historical data of its operations are necessary to obtain such results. In this sense, predictive maintenance has been introduced in many industries, not just as a warning system for possible future problems, but as a means of determining the cause of the problem, its severity, and the moment in time at which it may arise. It is also a way to design more effective solutions, by knowing where, how, and when they can fail. Furthermore, in predictive maintenance, an increase of sophisticated software that uses analytical modeling techniques has been observed. These techniques offer diagnostic information, prioritizing problems based on their severity and proposing measures to adopt for their prevention. In this way, human-caused errors can be minimized, maintenance costs can be reduced by 30% and downtime by 70% [7].

Safety should not be forgotten. Wind tunnels must also offer a controlled and safe environment for users and workers. Comfort and safety during flights must be guaranteed. The speed of the wind in the flight chamber requires a proper airflow distribution and velocity. Flight chambers can accommodate several flyers at a time, and their turbines can move up to 1.5 tons of air per second. It is very important that the reliability of the system is guaranteed during flights, including the air quality and prevention of turbulence. This study improves the control of the performance of such types of wind tunnel systems, placing great importance on user safety and the efficiency of the system. Therefore, it is necessary to correctly identify anomalous operations in any of the components that are part of the system—that is, both fans and their drives, as well as the tunnel as a whole—through the acquisition and monitoring of data that should be analyzed to extract valuable information.

Different kinds of sensors are used to collect wind tunnel system data. These sensors are located in different places, such as fans, drives, control cabinets, and the wind tunnel itself. Fan sensors have the role of capturing data related to the engine winding temperature, front- and rear-engine-bearing temperature, engine vibrations, and pressure. Drive sensors measure speed, current, power, voltage, IGBT (Insulated-Gate Bipolar Transistor) temperature, general temperature, and energy. Other sensors located inside the tunnel measure variables such as general system speed, total power of the system, total flow, air speed, and inside wind tunnel temperature.

Once we obtain the data from the sensors, the first objective is to determine whether the wind tunnel is running correctly and to identify if all data received are within normal parameters. This means that all of them must be within the usual range of values for that variable. If variable values are not within a specific range, it can be considered as an anomaly or outlier. To identify anomalies, data science methods can be used, specifically the Anomaly Detection Knowledge Unit (of the Data Mining Knowledge Area of the Data Analytics Knowledge Area Group) [8]; however, other methods can also be used, as pointed out in the second section. Anomaly Detection is a broadly used data science knowledge unit that has been profusely researched and applied in a wide variety of industrial sectors and research fields. Anomaly detection refers to the ability of a system to automatically detect anomalous values. An anomalous value is to be understood as an observation that deviates enough from other observations, such that it arouses suspicion that it was generated by a different event [9].

The need to detect anomalies and implement agile techniques for their identification is an open problem, and many detection techniques have been published in different fields of knowledge. Its popularity, especially in the industry, has promoted the development of specific tools for detecting outliers, in order to prevent errors and failures in systems [10]. In general terms, anomalies are patterns in the data that do not conform to well-defined normal behavior, ruling out data collection errors, sensor failures, and so on.

There are different methods of detection, which depend on certain aspects, such as the data available, the scope in which it is applied, and even the analyst who implements it. Further, in the detection of anomalous values, time is an interesting aspect; that is, anomalous behaviors in the data are examined over time. Unexpected changes and temporal patterns are used as mechanisms to identify and create models based on the detection of outliers [11]. Some authors have classified anomaly detection techniques into the following approaches: Distribution, Depth, Clustering, Distance, Density, Spectral decomposition, or Classification [12].

Depending on the kind of data available, we can talk about other means of detecting anomalies, such as Supervised, Unsupervised, and Semi-supervised methods. Most of these methods are detailed in the following section [13].

In many cases, due to the nature of the variables involved in a given study, it is possible that the application of a unique type of anomaly detection technique cannot solve the problem of the identification of outliers in all of them, such that different techniques must be applied, depending on the specific variable that is being studied. In other cases, even the application of different published techniques to different variables does not allow for obtaining the solution that is being searched for, due to the computational cost of their application or for other reasons. In such cases, a specific and adapted method must be developed, in order to solve the problem in the most efficient way. By merging both approaches, the best solution for the problem of anomaly detection can be solved in the best way. A method of this kind, composed of several methods, is usually called an Ensemble or Mixture Method or Model, where each one of these methods differs from the rest, depending on the primary methods used. The method is even more original if some of the primary methods have been developed specifically to solve the outlier detection problem in the studied system, as is the case in this study.

The remainder of this article is organized as follows: Section 2 discusses the theory of anomaly detection and time-series data. Section 3 includes an overall description of the proposed models defined by the combination of different techniques to define outliers from the use of the values obtained from the sensors in a wind tunnel, as well as the training approach for the model using a real database obtained from a set of sensors installed in a real machine built by a major company. Section 4 describes the research carried out to demonstrate the performance of the defined models using the same real database as in the previous section. Section 5 summarizes the conclusions obtained in the research and describes possible future works that can be done. Taking this research as a starting point, some of that research has already begun.

## 2. Theoretical Foundations

In this section, we present the theoretical foundations supporting the research. An anomaly or outlier is a datum from a set of data, sample, or population that is being analyzed, which is different enough from the rest of the data in the set to be considered uncommon. From this definition, a question arises: What does “different enough” mean? The methods developed in the field of data acience provide answers to that question and allow us to deal with the outlier identification problem.

This research uses a combination of three Data Science Knowledge Areas—Statistical Methods for Data Analysis, Machine Learning, and Data Mining—all belonging to the Knowledge Area Group Data Analytics—to address the problem of correct outlier detection in the data variables for wind tunnel systems.

To identify outliers, Data Science knowledge can be applied; specifically, the Data Science Knowledge Area Group Data Analytics, KAG1-DSDA [14] has three Knowledge Areas, KA01.01 (DSDA.01/SMA) Statistical Methods for Data Analysis, KA01.02 (DSDA.02/ML) Machine Learning, and KA01.03 (DSDA.03/DM) Data Mining. These knowledge areas provide different methods and techniques that can be used to deal with such problems. From KA01.01, the Knowledge Units used were KU1.01.06 (Exploratory and confirmatory data analysis) and KU1.01.07 (Quantitative analytics); from KA01.02, the Knowledge Unit KU1.02.03 (Unsupervised Machine Learning) was used; and from KA01.03, KU1.03.04 (Anomaly Detection) was used. Due to the diversity of the variables acquired by the sensors in this infrastructure, in this research, the Knowledge Areas and Units previously mentioned were used to solve the problem of identifying outliers among the data analyzed.

Starting from the knowledge Areas and Units stated above, the majority of Anomaly Detection Models could be classified, taking into account their theoretical foundations as follows:

- *Statistical Models*. Statistical Anomaly detection models are based on statistical knowledge [15]; that is, statistical techniques and models. There exist different approaches for the classification of this kind of method, but in this research, a new one is proposed based on the classification into two main groups:
  - *Based on magnitudes*. These kind of models are based on the values of statistical magnitudes, which must be calculated previously. The possible outliers in the data set are identified from these values. These include, for example, Box and Whiskers, based on the use of quartiles, bagplot, mean and variance, or Cochran test.
  - *Based on probability distribution*. These kind of models are based on the probability distributions of the analyzed variables, which must be obtained previously. The outliers are identified from those distributions. Different types of probability distributions can be analyzed to find outliers, such as Normal and Log-Normal. These include, for example, the Chi-square test, Dixon test, Gribs test, Tietjen–Moore, or Generalized Extreme Studentized Deviation.
- *Distance-based Models*. These models are based on the definition of a distance function between the data, such as the Minkowski, Euclidean, Manhatthan, Mahalanobis, or Chebychev distance [16]. The distance is calculated between all the observations in the data set, and the values obtained must follow a certain pattern if the data are normal. The outliers are those values that do not follow this pattern [17,18]. These methods include, for example, K-nearest neighbors and Angle Bases Outlier Detection (ABOD).
- *Density-based Models*. These models are based on the amount of observations found in the different regions of the space. If the values are normal, the density must follow a pattern in different regions. If some regions appear with lesser density than the pattern observed, values in that region could possibly be outliers [19]. These include, for example, Local Outlier Factor (LOF) and Shared Nearest Neighbour (SNN).
- *Spectral Decomposition Models*. These models divide the data into sub-spaces of lower dimensions than the initial one and identify those sub-spaces as having normal, anomaly, or noise Sub-space Outlier Degree. Once these sub-spaces have been defined, outliers can be identified in the anomaly sub-space [20].

Another perspective to classify anomaly detection is based on the type of available data [21]. If some of the data to be analyzed can be classified as normal or outliers, the outlier identification approaches can be classified as follows:

- *Supervised Models*. These are models that use a training set that contains normal data and anomalies. If there are different classes of anomalies, they must be contained in the training set data.
- *Unsupervised Models*. These models are used when it is not known whether the data contained in the data set are normal or outliers; that is, if the data does not have a class label identifying whether the data is normal or an outlier. To establish whether a datum is an outlier or not, a parameter called the outlier *degree* is used. This parameter is set by the analyst and, for each datum, it establishes whether it is an outlier or not.
- *Semi-supervised Models*. In these models, some data in the data set are considered normal, while other data are not labeled as normal or outlier. These models use the labeled data as reference, in order to obtain a parameter that allows for the identification of the non-labeled objects as outliers or normal data.

The goal of this study is to develop a model that allows for the identification of outliers in the variables of wind tunnel systems with the lowest time and resource consumption possible, in order to ensure its application in real-time monitoring. For this reason, of all of the models previously described, statistical models based on probability distributions were selected to perform the identification of anomalous values within one of the two kinds of variables identified for the wind tunnel systems—those which do not present smooth behavior—as this kind of model allows for a two stage analysis. In this analysis, the normal range of values is established in the first step; in the second step, starting from the obtained range, the outliers can be easily identified as values that are outside that range, with low time and resource consumption. For the other kinds of variables—those that present smooth behavior—a model that fits the requirements of the desired exactness and performance was not found. Therefore, we developed a new method for obtaining the outliers based on distances. In the following subsections, the differences between these two kind of variables and the two approaches that constitute the proposed Mixture Model for the detection of anomalies in wind tunnels are described in detail.

### 2.1. Statistical Probability Distribution Outlier Detection Models

One of the most common statistical approaches used to identify outliers is the data probability distribution method. The data contained in the sample set are used to develop a probability distribution model for the studied variable. Once the model is obtained, it is used to identify data that are far from fitting the model; that is, data for which the probability of occurrence is far from being considered normal. From this point of view, an outlier is a value of a variable that has a very low probability of occurrence (if not zero), when we calculate it from a probability distribution obtained from the data set to which the data belong.

With the above in mind, the first step in using this method is to identify the specific probability distribution that fits the analyzed data set. This action must be done correctly, since if an incorrect distribution type is selected, the outliers will be incorrectly identified. There are specific density functions that are well known, as they appear many times during the process studied. This was the case of the density functions obtained from the data of the variables studied in this research using probability distributions. They are the Normal (or Gaussian) distribution and the Log-Normal (or Galton) distribution. Once we had obtained the values of our data, we defined a point to be an outlier, $x_{o}$, if its probability of occurrence had a value equal or lower than a defined value, $d_{o}$, known as the *outlier degree*.

### 2.2. Local Transilience Outlier Identification Method (LTO)

Most of the main methods used to identify outliers (i.e., those based on distances, density, or clustering) are based on the calculation of distances. These distances are calculated using a distance function such as the Euclidean, Manhattan, or Chebyshev distance. These approaches use different algorithms to obtain the outliers; however, in all of them, there is a common step: the calculation of distances between all of the points in the data set. This procedure has two main problems that make their use in the present case very difficult, if not impossible, as the identification of outliers in real-time is required, as well as only using the current data. These problems are, first of all, that data are needed to apply the method and, secondly, that a high computational cost is incurred (which is related to the previous problem).

The problems pointed out previously are common when time-series are analyzed, and different approaches have been published for solving them [19,22,23]. The common characteristic of most of them is that their algorithms present one or more steps in which a classification model (e.g., a regression model) must be calculated before addressing the same problem found in the other, more general, outlier identification methods.

Another difficulty for the application of the current methods is the determination of the outlier degree, for which previous samples must have been analyzed before or the current sample must be analyzed completely. If the outlier degree has been established by analyzing another sample, we must be sure that inference can be done and that the current sample belongs to the same population; if the outlier degree is being determined for the current sample, we need to finish the analysis of the whole sample first and cannot establish the outliers in real-time.

Depending on the features of the variable that is being analyzed, the problems pointed out previously cannot be avoided, and any of the listed methods must be used to identify outliers in those variables. However, for a certain kind of variables, which we name laminar variables, a new method can be used, which allows for the identification of outliers in real-time with low computational cost. We name the proposed new method the Local Transilience Outlier Identification Method (LTO).

The idea of this new method for outlier identification is based on the study of fluid dynamics: when the fluid flow is laminar, the transmission of physical magnitudes, such as momentum or energy, between layers is done gradually, such that the change in the gradient observed for those magnitudes is smooth. However, when chaos arises and the flow becomes turbulent, the interchange of energy or momentum between layers that are at some distance can be observed, which is called turbulent transience. The analogy that allows for the application of transilience to the problem of outlier identification is that the normal behavior for some kinds of variables that can be found in different types of fields of study in normal conditions is inherently smooth (or laminar); that is, depending on their definition reference system, the gradient is quite linear in space or in time, without significant leaps between values. If the behavior of one of these variables becomes turbulent in any moment or place, a large change will be observed at the moment or place in which the turbulence appears.

For variables whose usual behavior is not laminar, this method cannot be applied. However, once a variable has been cataloged as laminar, the local transilience outlier identification method (LTO) can be applied in order to identify its outliers in a rapid and computationally efficient manner [19]. The term Local refers to the fact that only the nearest (spatial or temporal) neighbors are needed to identify the outliers. Moreover, to be applied locally, a second important characteristic of the LTO method is that it does not use the values of the variable analyzed for identifying the outliers but, instead, uses new derived values, such as the mean of the difference of values between the value of the variable and the values of their nearest neighbors. To measure the difference between values, several approaches can be used. In this paper, two such approaches are covered: the Euclidean distance for two-dimensional variables and the arithmetic difference for one-dimensional variables. However, other approaches could be proposed to better fit each specific problem studied.

For two-dimensional variables, the proposed method operates in two main phases:

- In the first phase, the *laminarity* of the studied variable is established in the following way:
  1. The local area is established. The number *k* of nearest neighbors that will be used to perform the study is established: k=3 or k=4 may be enough, but lower or greater values can be selected. In this paper, k=3 was used.
  2. For each value of the variable *v* at time *i*, $v_{i}$, the Euclidean distance to the *k* nearest values is calculated:
    (1) $$forj=1,k;d_{i,i-k}=\sqrt[{}]{\sum_{i=1}^{ds}{\left(v_{i}-v_{j}\right)}^{2}},$$
    where the sum is from 1 to ds, depending on the number of dimensions of the variable *v* (in this study, ds=2 or ds=1); and k depends on the value selected in the previous step (in this study, as k=3, we use $v_{i-1}$, $v_{i-2}$, and $v_{i-3}$).
  3. Once the three distances are obtained, the second step consists of the calculation of their mean:
    (2) $$m_{i}=\frac{\sum_{j=1}^{k}d_{i,i-j}}{k}.$$
    This is performed for all values in the training set.
  4. The third step consists of the calculation of the mean and variance of the means calculated in Step 2. If there are *n* values in the data set, the calculation is:
    (3) $$\begin{array}{cc} m=\frac{\sum_{i=1}^{n}m_{i}}{k} & {\sigma_{m}}^{2}=\frac{\sum_{i=1}^{n}{\left(m_{i}-m\right)}^{2}}{k}. \end{array}$$
    Once *m* and var have been obtained for the training set, they are used to identify anomalies in the variable for which they have been obtained during the normal operations of the laminar variable (in the case of this paper, wind tunnel variables), by applying a statistical method based on probability distributions (as explained in Section 2.1) to obtain the interval for normal $m_{i}$. This interval is defined as the degree of non-laminarity of the value. In the case of this study, the interval range was established as $\pm 3\sigma^{2}$
- In the second phase, the same three first steps of Phase A are applied to the sample under study, and $m_{i}$ for each one of them is obtained. To detect outliers for each observed value, we check whether it is within the laminarity interval or if, on the contrary, the mean leap value is outside the said interval; in this case, it is classified as a transilent leap. This can be done in real-time as, for each value, only the value of the *k* nearest (or, in the case of time-series data, the *k* previous) values of the variable are needed.

In consequence, if a value in the sample is inside the interval, the value is considered normal; however, if it is outside the range, the value is considered an outlier. This method requires a low computational cost, and when it was applied to the data used in this study (see the following sections), it was shown that it is very efficient in detecting outliers for laminar variables (i.e., those that have a smooth evolution over time), as outliers are not identified for each value but, rather, by the sudden change in their value, not following the logical and normal evolution of the variable.

If applied to time-series, the proposed outlier detection method only takes into account the non-smooth leaps in the previous values of the analyzed data for the detection of anomalous values, while the subsequent values are not taken into account for the analyzed value. The reason for this is that, once an outlier has appeared, its value may remain constant in the following *j* observations of the variable. In this case, if the method were bilateral, the right tail could muffle the observation on the left, thus leading to incorrect outlier identification.

The Local Transilient Outlier method (LTO) algorithms are as follows (Algorithms 1 and 2):

- In Phase A, establishing the laminarity of the variable and the range of normal values.

**Algorithm 1:** Establishing the laminarity of the variable and the range of normal values.
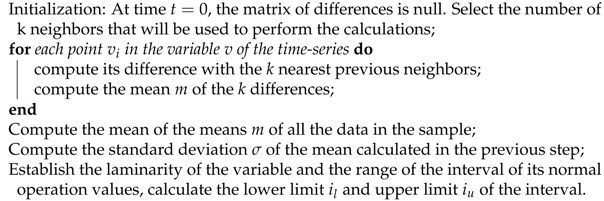

- In Phase B, the LTO method is used to detect the outliers of the variable.

**Algorithm 2:** Using the LTO method to detect the outliers.

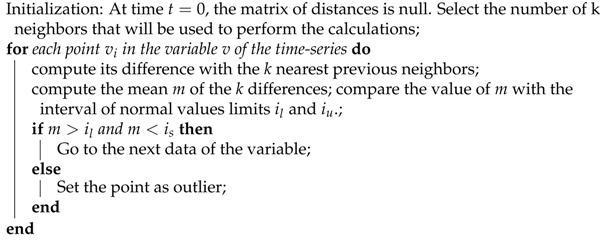



## 3. Anomaly Detection in Wind Tunnel System Performance

Based on the theoretical foundations presented in the previous section, we carried out further research, the results of which are presented below. The study has two main parts. In the first, all the variables that intervene in the operation of the machine were analyzed, in order to develop a model for detecting anomalies in each of them. To do this, different approaches were used to detect outliers, taking into account the nature of each of them. Once all the models were obtained to identify anomalies for each variable, a study of the wind tunnel as a whole was carried out and a classification model was developed, in order to be able to analyze the set of values that produce anomalies (in terms of the performance of the system), and whether or not the global anomaly of the wind tunnel is related to anomalies in its different parts.

### 3.1. Variables Used in Monitoring the Behavior of Wind Tunnels

The type of wind tunnel analyzed in this study, among the existing types, was a vertical one. It is composed of four axial fans with a diameter of 3400 mm, accompanied by four drivers, PLCs (Power Line Communications), a control desk, and several sensors that collect the information necessary for monitoring the system. The four fans work synchronously in a system called a “double loop”; that is, two fans are placed in parallel on both sides of the flight chamber, two in each loop. The system causes the air flow to converge in a central duct, where the flight chamber is located. The total power installed is 1.400 kW. The system is designed such that the fans have the same speed and, so, the speed setpoint is digital, thus ensuring that the setpoint requested by the flight operator is the same for the four drives.

**Figure 1:**
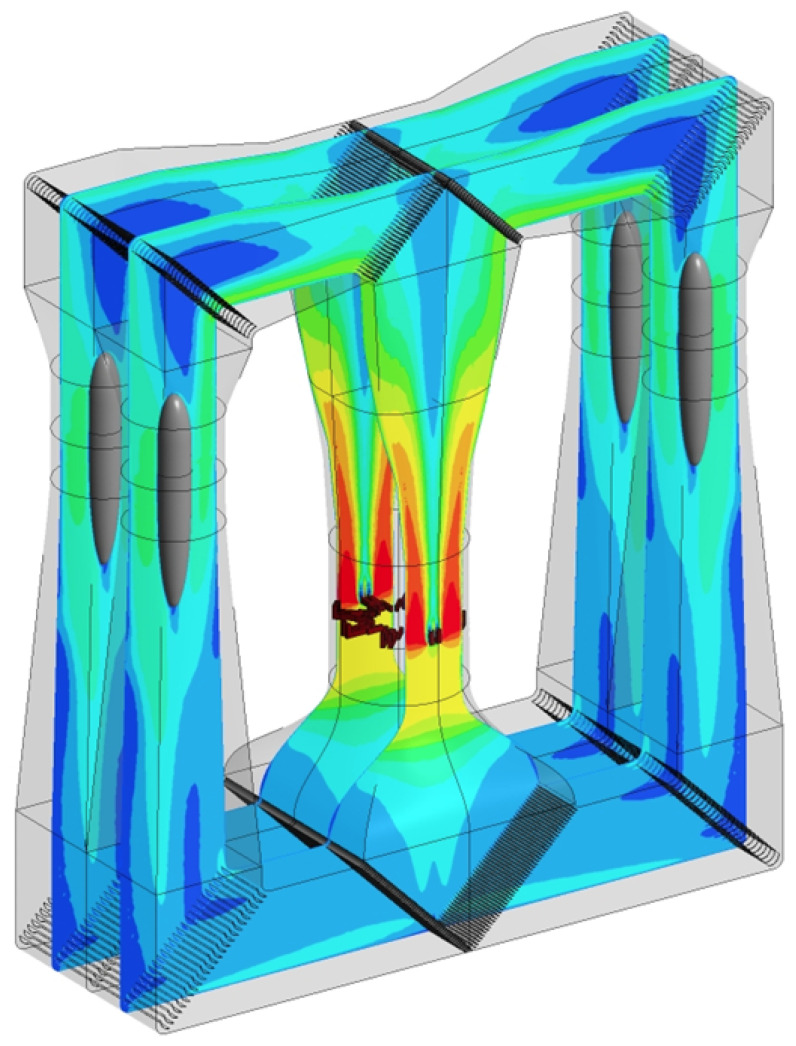
Computational Fluid Dynamics (CFD) simulation for a double-loop wind tunnel.

Figure 1 presents a CFD (Computational Fluid Dynamics) simulation of a double loop wind tunnel. Its structure described above allows air to circulate at both sides of the tunnel, in order to finally reach the flight chamber (i.e., the space where users fly). The use of Computational Fluid Dynamics (CFD) simulations can provide insights to help with the optimization of the wind tunnel design.

**Figure 2:**
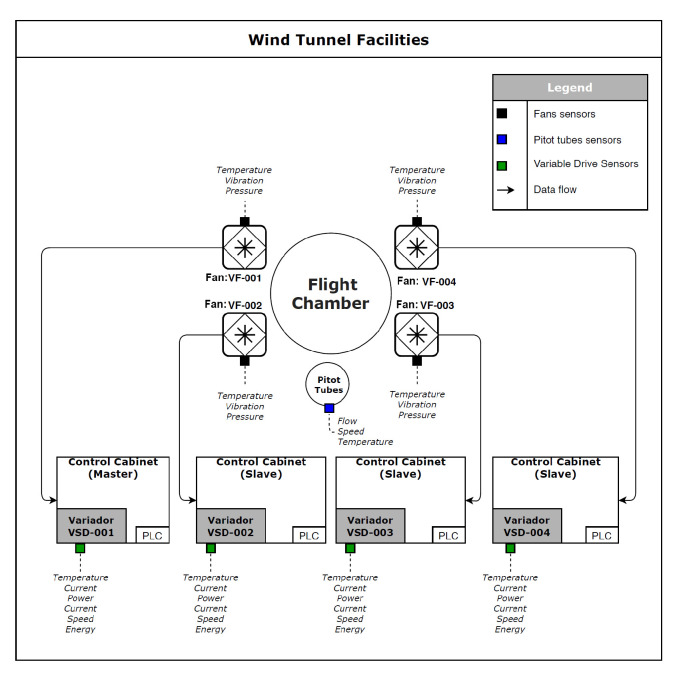
Wind tunnel system diagram.

Figure 2 presents the general operational workflow of a wind tunnel, detailing the main components and their connections. Each of these fans have drivers to capture data from the fan and from their own driver. Additionally, the fans have sensors located in the engine and fan casing. The monitoring and data acquisition systems set up in this facility collect data every minute, each time an event takes place, and when facility operators establish the speed or time in the system for each flight session. Therefore, a large number of data are generated by the system. For our analysis, a database with a total of four different collections of data were used. One collection contained data about events, such as warnings, alarms, events, and commands. Another collection of data focused only on fan variables, while another collection contained data about fan drivers, and, finally, the other collection was data regarding the general variables of the facility. The collection and storing data systems established a ModbusTCP connection with the control PLC, managing different tasks such as communication between machine sensors and the system, data collection, post-processing of captured data, and real-time data display.

Each axial fan propels air through a propeller that has several blades, usually constructed of carbon fiber, that are anchored to a core from which the energy comes. They are designed taking into account a series of parameters, such as revolutions per minute, diameter, flow, and pressure, among others. Likewise, the number of vanes, their angle, and width are determined by the system requirements, as the result of engineering work that provides a tailored solution for that specific installation. The speed driver in this type of installation has the objective of regulating certain aspects of the fan, such as its frequency, thus controlling the speed at which the motor rotates. Finally, the control desk is the general control memory, providing a general entity that groups certain parameters of the installation system.

As mentioned previously, in each part of the wind tunnel, there are different sensors that play very important roles for the safety and efficiency of its operation. They are located in fans, drives, and in the general tunnel installation, with the purpose of collecting data of each variable involved. The installed sensors are used to monitor the system behavior, but are also to support the remote control of the performance of the wind tunnel elements.

This type of facility could have from 35 to 70 sensors that collect and submit information to the system where data are stored, analyzed, and reported through a dashboard, collecting information every 60 s and every time a manual action from a controller at the console on the flight deck takes place. In this way, the real-time status of the system can be monitored. The software is also equipped with alarm features, which send information about the performance of the system. On average, 18,000 measurements are collected on a daily basis. Due to the large number of data generated from the system, time-series segmentation is critical for time-series analysis.

The airspeed in the tunnel chamber changes depending on the number of flyers. This is set up manually by the controller, who precisely determines the level based upon the number of flyers, their weight, and their skill. However, there are also other variables that use sensors to optimize and secure the flight experience. For example, the four fan motors have vibration sensors. These sensors are in charge of monitoring fan/motor vibration, to detect abnormal levels, and to reduce the airspeed or stop the system. Other important sensors are the air pressure sensors, which measure the wind speed and efficiency of the system. These are commonly pitot tubes. There are also temperature sensors, which measure different aspects such as flight chamber temperature, driver temperature, and motor bearing and winding temperature. Other sensors located in the system measure current, flow, power, or energy.

A data acquisition system was developed to collect inputs from various sensors, transferring the data to a point where the data could be accessed for further analysis. The system establishes a ModbusTCP connection with the control PLC, in order to manage the tasks described below:

- Communication set-up.
- Acquisition of data.
- Real-time visualization of the installation status with Real Time System.
- Information storage in databases with a Reporting Server System.
- Post-processing of the information with Reporting Server for later historical visualization through graphics using the Real Time system.

The variables measured by the sensors were of two types:

- Analog. These can take a continuous value, usually within a known range. This is the case of the air speed, which is a percentage value of the operating setpoint, which normally takes values between 0 and 100%. In this case, it could be a minimum limit programmed in the PLC. The maximum value responds to the value configured for the application, which, in this case, is greater than 100%, such that it was configured with the possibility of operating at overspeed.
- Digital. These are bi-state variables, such as YES or NO, ONE or ZERO, TRUE or FALSE. A ZERO/FALSE value indicates that the event is not active. A ONE/TRUE value indicates that the event is active. If there is a TRUE in a run command, it means that the system is activating the run command. If there is a TRUE in an alarm, that alarm is active; when it goes to FALSE, the alarm stops—this could be due to a condition or acknowledgment. CONDITION means that if the condition that produces the alarm disappears, the alarm event also disappears. ACKNOWLEDGMENT means that if the condition that produces the alarm disappears, the alarm event does not disappear, such that an acknowledgment action by the operator is necessary (e.g., pressing an alarm reset button). The capture of event data allows for identifying the number of alarms produced and their typology. Events can identify previous malfunction situations, so their analysis can allow for anticipating a machine stoppage or the performance of preventive maintenance actions.

Data are collected and stored for analysis on a daily basis every time an event occurs or every 60 s. Among the data collected by the sensors, there are data related to events such as operation alerts and data of specific variables, such as temperature, speed, and vibrations.

The capture of event data allows us to determine the number of alarms produced and their type. The events can identify previous situations of malfunction, such that their analysis could lead to anticipating the shutdown of the machine or the performance of preventive maintenance actions.

Among the events collected by the monitoring system, special interest should be given to the data regarding warnings and failures. Some of them are warnings related to temperature, pressure, speed, or vibrations, which are collected every time such an event takes place.

The variable data from the wind tunnel fans, together with the variables from the drivers and from the installation as a whole, allows us to perform a detailed analysis of the overall operation of the system.

The variables measured, regarding the wind tunnel fans, were as follows:

- Fans:
  - Speed. Fan rotation speed. Measured in percentage (%); as a consequence, the expected value is in the interval (0, 100). It is measured by the drivers.
  - Speed reference VSD (Variable Speed Drives). Speed reference for the drivers. This is the speed setpoint of the driver, which indicates that its value is a percentage; as a consequence, the expected value is in the interval (0, 100). If the driver is receiving a setpoint of 50%, it is modulating its nominal output. It is measured by its own driver.
  - Current. Average instantaneous current of the motor output phases. Measured in Amps (A). It is measured by the driver.
  - Power. Instantaneous power consumed by the motor. Measured in Kilowatts (kW). It is measured by the driver.
  - Winding temperature of the motor phases V, U, and W. It is a three-phase motor and this variable measures the different winding temperatures of the motor for phases V, U, and W. This is measured in degrees centigrade (ºC), measured by sensors installed in the motor winding. A value of 850 ºC indicates a problem in the probe or in the acquisition electronics.
  - Engine front and rear bearing temperatures. These are measured in degrees centigrade (ºC), and are collected by sensors installed in the motor bearing. Sensors read by ZMDAQ electronics, PT100 input channel. A value of 200 ºC indicates a problem in the probe or in the acquisition electronics.
  - Vibration in the general motor. Measured in millimeters/second (mm/s). It is measured by sensors installed in the motor housing. Sensor reading by ZMDAQ electronics, analogue input channel 4.20 mA.
  - Measured pumping pressure value. Measured in Pascals (Pa). It is measured by a Petermann sensor in the fan. The pressure differential between taps B+ and B- is measured using a pneumatic inlet channel.
- Drivers:
  - Alternating voltage between the driver input phases R and S.
  - Alternating voltage between the driver input phases S and T.
  - Alternating voltage between the driver input phases T and R.
  - Average alternating voltage of the three motor phases U, V, and W
  - Motor output IGBT temperature (these are the most critical switching elements).
  - General temperature of the motor.
  - Instantaneous power output to motor.
  - Average current of the three motor phases U, V, and W.
  - Motor output phase U current.
  - Motor output phase V current.
  - Motor output phase W current.
  - Instantaneous speed of motor rotation in percentage.
  - Instantaneous speed of motor rotation in Hertz.
  - Instantaneous speed of motor rotation in revolutions per minute.
  - Sum of energy consumed; these are data returned by the drive.
  - Sum of energy regenerated to the grid; these are data returned by the drive.
  - Instantaneous drive input power.
  - Inverter input phase R current.
  - Inverter input phase S current.
  - Inverter input phase T current.
  - Cosine of Phi at the input of the drive.
  - Harmonic distortion value in intensity at the input of the drive.
- Wind tunnel facility:
  - System speed setpoint in percentage.
  - Sum of powers of the four fans.
  - Total flow value of the installation.
  - Air flow speed.
  - Temperature setpoint inside the tunnel.
  - Measurement of the temperature inside the tunnel.

### 3.2. Anomaly Detection in Wind Tunnel Variables

The first part of this study was to establish anomaly detection models for the most important variables involved in the performance of the Wind Tunnels. Two different approaches were used, depending on the variable studied: statistical methods based on probability distributions and the Local Transilience Outlier Identification Method (LTO).

#### 3.2.1. Applying Modified Local Transilience Outlier Identification Method (LTO) for Outlier Detection in Wind Tunnels

In the group of variables analyzed, there was a subset composed of variables for which an appropriate approach for identifying outliers was the use of the proposed Local Transilience Outlier Identification Method (LTO). The reason for selecting and applying this method to these variables was that we were dealing with variables with specific behavior: their value can only increase and decrease in a smooth manner. For this reason, considering how we obtain their values from the sensors each minute, at a specific time, the value of the variable must be related to the previous and following values and, in consequence, if the value measured at that time is very different from the previous values, that value could be considered an outlier. A similar approximation for identification of anomalies in time-series data has already been used in other similar studies [24].

As was described in the previous section, to use the Local Transilience Outlier Identification Method to solve an specific problem of anomaly detection, two main parameters must be defined: the interval of correct operation (i.e., the range at which a value can be considered too far from the others and, so, can be considered an outlier) and the *k* neighbors to which the distance calculation will be applied. In this problem, the values of each measure, if the machine is running correctly, must be related to the previous ones and, to avoid the problem of correctly selecting *k* such that a failure in the measurement of an specific instant by the sensor will not lead to identifying a problem that that does not really exist, it is better to introduce a modified version of the method, in which it is not applied to the distance with a specific value of *k* but, instead, over the mean of the distance of each value with the three previous values measured; this is the LTO model.

The LTO model takes only the three previous values and not the subsequent values as, if an anomaly appears, it may be maintained in time for some period (e.g., three minutes), and, if we take the values measured on those moments to determine the mean, the distances obtained may be close to zero and the outlier could be hidden. The reason for using only three data points is because, with three values, the problems derived from using only one value can be avoided while the cost of computation can be kept low. In this way, this method can be implemented in a real-time system.

#### 3.2.2. Applying Probability Distributions for Anomaly Detection in Wind Tunnels

In the group of variables analyzed, there was another subset composed of variables for which an appropriate approach for identifying outliers was the use of probability distributions. The reason for not having selected the LTO method for these variables, as for the other set of variables, is that very different measures can be found between correlative values of those variables and, if we used the distances between them, we could obtain distances over the outlier degree for values that were absolutely normal. For example, we could have a pressure of 50 and the following value of −60; if we established k=1 and a interval of normal values of 100, the value of −60 would be identified as an outlier despite being perfectly normal.

For pressure, the observed data set was composed of 44,000 values. Those values belonged to the four ventilators, and the first step performed for their analysis was to separate the set into four subsets, one for each ventilator. The resulting subsets were $p_{vi}$, with i=(1,2,3,4), where the size of each subset was the same (11,000 data). Once we obtained the four subsets, we divided each of them into two, one for training tr and the other for testing tt, where 8000 data were used for the training set and 3000 were used for the testing set. Finally, we obtained eight sets, $p_{vi-tr}$ and $p_{vi-tt}$, with the same *i* (from 1 to 4).

For vibration, we carried out the same steps as for pressure, obtaining the four following training and test sets: $vr_{vi-tr}$ and $vr_{vi-tt}$, with the same *i* (from 1 to 4).

## 4. Research Results

In this section, a summary of the research results, along with the new findings for which the research has been done, are presented. In the next subsection, we present the intervals of correct operation and anomalies observed in the variables involved in wind tunnel performance obtained from the research. In the second part of this section, the same results are presented, but after the classification was performed and the wind tunnel as a whole was studied.

### 4.1. Intervals of Correct Operation and Anomalies Observed in Variables Involved in Wind Tunnels Performance Applying the Probability Distribution Method

This section presents the results obtained from the data analyzed for each of the variables studied. The figures included in this section show outliers found during the analysis of the different variables. All data found outside the normal range are represented by red dots in the plot. The density represents, in an effective way, the distribution of the variable analyzed.

The variables studied under the model based on statistical probability distribution outlier detection methods were as follows:

- Pressure: The values showed a marked difference for one of the fans, where the mean values and standard deviation were above those of the rest of the fans. The fans reached very different minima and maxima, and it is necessary to remember the description of the pressure variable previously made, where it was specified that the fans should operate normally at a negative mean value (or close to zero). There was a limit value of 400 Pa, above which the fan operates in a high pump zone. Above this value, one should could consider stopping its operation. Therefore, the pressure distribution analysis allowed us to observe frequent values above zero and even exceeding 400 Pa. Although the distribution curve resembled a Gaussian distribution, it can be seen that, in most cases (except for fan 1), although there was symmetry with respect to the mean, the means had positive values, the highest being that of fan 3. The data obtained during the analysis allowed us to see the intervals detected by the model, where the normal operating values would be. Any value outside this range would be an anomalous value within the operations of the system. With the developed method, a range of normal values was established, in which 99.99% of cases were found.
  - #1 Fan: −152.4557  140.9509
  - #2 Fan: −152.9021  166.1269
  - #3 Fan: −220.8516  250.6594
  - #4 Fan: −140.6571  159.1673
  Running the probability distribution method on the test data yielded the following results:
  - #1 Fan: 0.4% of outlier values were detected, exceeding mainly positive values.
  - #2 Fan: 0.7% of outlier values were detected, exceeding mainly positive values.
  - #3 Fan: 0.6% of outlier values were detected, exceeding mainly positive values.
  - #4 Fan: 0.5% of outlier values were detected, exceeding mainly positive values.

**Figure 3:**
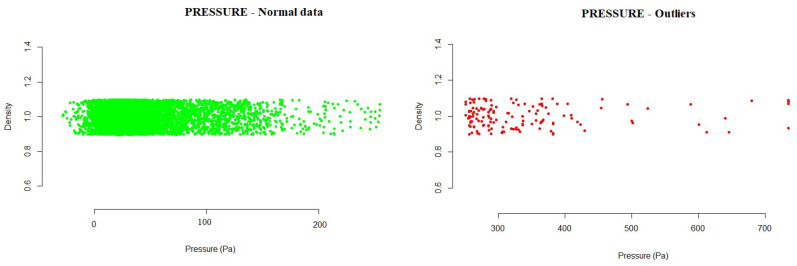
Example of pressure outliers detected for one of the fans in the testing data.

Figure 3 shows the outliers found during the analysis of the pressure. Outside the normal range calculated were those values higher than 250.6594, as can be seen in the graph.

- Vibration: This refers to the vibration in the general engine; it is measured in millimeters per second, where values above 10.2 mm/s are excessive. The values were collected through a sensor in the motor housing.
  The vibration variable was analyzed to identify anomalous values. The following data present the intervals detected by the model where the normal operating values would be. Any value outside this range would be an anomalous value within the operations of the system. With the developed method, a range of normal values was established, in which 99.73% of cases were found.
  - #1 Fan: −1.365203  4.060265
  - #2 Fan: −1.287130  3.253275
  - #3 Fan: −1.384863  2.924214
  - #4 Fan: −2.814419  6.267012
  Running the probability distribution method on the test data showed the following results:
  - #1 Fan: 0.3% of outlier values were detected, exceeding mainly positive values.
  - #2 Fan: 0.1% of outlier values were detected, exceeding mainly positive values.
  - #3 Fan: 0.08% of outlier values were detected, exceeding mainly positive values.
  - #4 Fan: 0.01% of outlier values were detected, exceeding mainly positive values.

**Figure 4:**
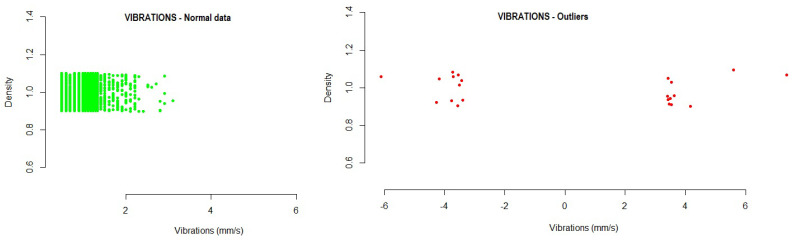
Example of vibration outliers detected for one of the fans in the testing data.

Figure 4 shows the outliers found during the analysis of the vibration variable. Those values calculated to be outside the normal range are represented as red dots in the graph.

**Figure 5:**
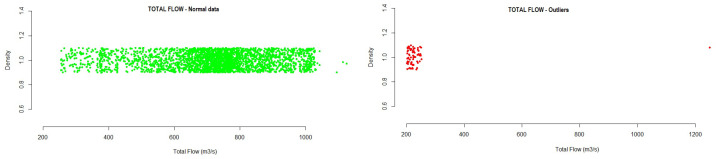
Example of total flow outliers detected for one of the fans in the testing data.

**Figure 6:**
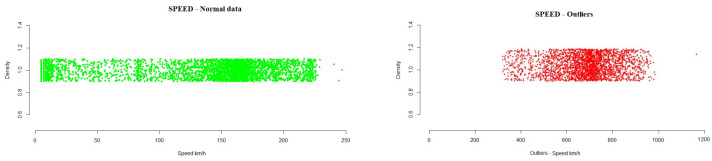
Example of speed outliers detected in the testing data.

**Figure 7:**
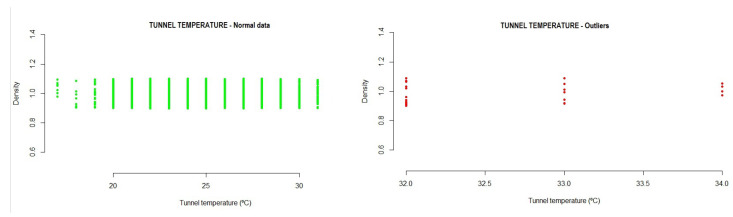
Example of tunnel temperature outliers detected in the testing data.

- Flow: This is a variable relative to the installation, which represents the total flow value of the installation, measured in m${}^{3}$/s. Differential pressure is measured in the installation, in order to calculate the flow based on that pressure with the measurement section configured in the PLC. From the analysis of the variable total flow, the following interval was obtained, where the normal operations of the system must be located.
  - Total Flow: 251.8248  1159.6980
  During the execution of the analysis method, the test data showed that a total of 0.9% outlier values were detected, while 98.75% of the data were in the range of normal values, as can be seen in Figure 5.
- Speed: This is the air flow speed, in km/h, which is calculated based on the differential pressure measurement of the installation.
  From the analysis of the variable speed, the following interval was obtained, where the normal operation of the system must be located.
  - Speed: −30.94886  291.87405
  During the execution of the analysis method, the test data showed that a total of 0.3% outlier values were detected, where 98.70% of the data were in the range of normal values, as can be seen in Figure 6.
- Temperature: This is a measure of the general installation, in terms of the interior temperature in the tunnel. It is measured in degrees centigrade and consists of values that a cooling device sends to the system. From the analysis of the variable temperature, the following interval was obtained, where the normal operation of the system must be located.
  - Temperature: 15.90087  31.51146
  During the execution of the analysis method, the test data showed that a total of 0.3% outlier values were detected, where 99.70% of the data were in the range of normal values, as can be seen in Figure 7.

### 4.2. Intervals of Correct Operation and Anomalies Observed in Variables Involved in Wind Tunnel Performance Applying the New Mean of Distances to Local Transilience Outlier Identification Method (LTO)

**Figure 8:**
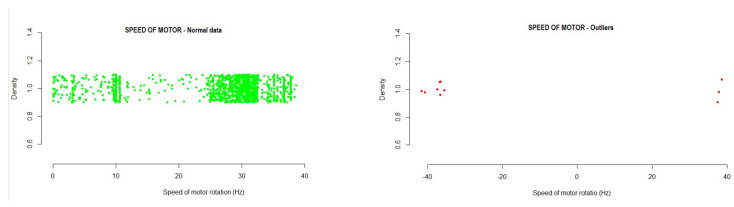
Example of the instantaneous speed of motor rotation outliers detected in the testing data.

**Figure 9:**
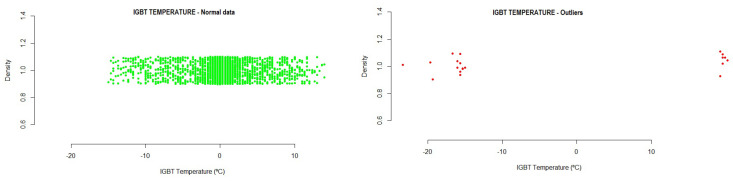
Example of the Insulated-Gate Bipolar Transistor (IGBT) temperature outliers detected in the testing data.

The variables studied under the method based on the new mean of distances to LTO outlier identification method were as folows:

- Speed: This variable is found within the data collection of the fan frequency drivers and collects the instantaneous speed of motor rotation in Hertz, a value that is returned by the driver itself.
  The data obtained in the analysis allowed us to identify the intervals where the normal operating values would be. Any value outside this range would be an anomalous value within the operations of the system. With the developed method, a range of normal values was established, in which 99.99% of cases were found.
  - #1 Driver: −35.60271  35.61316
  - #2 Driver: −35.70871  35.71897
  - #3 Driver: −35.16069  35.17099
  - #4 Driver: −35.19508  35.20562
  Running the method based on the new mean of distances to LTO outlier identification on the test data showed the following results:
  - #1 Driver: 0.1% of outlier values were detected, exceeding mainly positive values.
  - #2 Driver: 0.2% of outlier values were detected, exceeding mainly positive values.
  - #3 Driver: 0.2% of outlier values were detected, exceeding mainly positive values.
  - #4 Driver: 0.1% of outlier values were detected, exceeding mainly positive values.
  Figure 8 shows the outliers found during the analysis of the variable. Those values outside the normal range (and, thus, identified as outliers) can be seen in the graph.
- Temperature IGBT: This is the temperature of the motor output IGBTs, which is a very critical element in the system. The temperature is measured in degrees centigrade and is a value returned by the drive. The data obtained in the analysis allowed us to identify the intervals where the normal operating values would be. Any value outside this range would be an anomalous value within the operations of the system. With the developed method, a range of normal values was established, in which 99.99% of cases were found.
  - #1 Driver: −16.13426  16.13375
  - #2 Driver: −16.21591  16.21481
  - #3 Driver: −16.27438  16.27396
  - #4 Driver: −14.84254  14.84203
  Running the method based on the new mean of distances to LTO outlier identification on the test data showed the following results:
  - #1 Driver: 0.2% of outlier values were detected, exceeding mainly positive values.
  - #2 Driver: 0.3% of outlier values were detected, exceeding mainly positive values.
  - #3 Driver: 0.3% of outlier values were detected, exceeding mainly positive values.
  - #4 Driver: 0.3% of outlier values Were detected, exceeding mainly positive values.
  Figure 9 shows the outliers found during the analysis of the variable. Those values outside the normal range (and, thus, identified as outliers) can be seen in the graph.

## 5. Conclusions and Future Work

This study presented a solution focused on optimizing the operations of wind tunnels with the characteristics described in this research, thus improving engineering and performance aspects. The aim was to monitor the behavior of a flight chamber in order to detect possible anomalies in its operation. In the flight chamber, different sensors are deployed in critical elements in order to ensure its safe operation. The wind speed in the flight chamber requires an appropriate airflow distribution and velocity. The flight chambers can accommodate several flyers at a time, and their turbines can move up to 1.5 tonnes of air per second. The reliability of the system is very important to ensure flight safety and turbulence-free air quality. For its solution, an ensemble method was presented, in order to analyze the different variables captured from the flight camera. This method was mainly composed of two primary anomaly detection methods, one of which analyses a type of variable that we call laminar variables. The laminar property is typical of fluid dynamics, where the exchange of physical quantities (e.g., momentum or energy) between layers is performed gradually, with the magnitude of the observed gradient being smooth. For the analysis of anomalies of this type of variable, the authors presented a new method, called Local Transilience Outlier Identification Method (LTO), which allows for the analysis of anomalies of this type of variable in real-time and at low computational cost (as required for this type of system). In this work, outliers are defined as the malfunctioning of one of the critical components of the wind tunnel; however, it is likely that an outlier could be due to the sensor itself. In this case, and due to the criticality of the system, the team of engineers in charge of the maintenance of the installation must check the operations of all the equipment, including the sensors.

In this research work, a proof-of-concept was carried out on a real installation, and the reliability of the proposed anomaly analysis method was tested. This ensemble method provides a new LTO algorithm, which allows for the analysis of laminar variables in both temporal and spatial dimensions and can be applied to any other optimization process for similar systems. For the development of the proof-of-concept, real data from an installation were used. The results obtained from this ensemble method were validated by the installation’s engineers. From their point of view, in consideration of the results obtained by the ensemble method, the anomaly detection method can be considered satisfactory. Although it is true that other methods could be proposed to solve the problem, the engineering team positively valued the results obtained, above all respecting the need to operate in real-time with limited computational resources. In fact, it is now the method used to detect anomalies in the considered wind tunnel. However, in order to analyze optimizations for the proposed solution, we propose the research of other methods for solving this problem as future work. As additional future work, the authors are working on demonstrating the properties of the LTO algorithm for its application in other engineering challenges, where both spatial and temporal dimensions must be taken into account.

## Data Availability

Restrictions apply to the availability of these data. Data was obtained from private company and are not public available.
